# Histone Deacetylase Inhibitor (SAHA) Reduces Mortality in an Endotoxemia Mouse Model by Suppressing Glycolysis

**DOI:** 10.3390/ijms241512448

**Published:** 2023-08-04

**Authors:** Yunchen Wu, Yudan He, Chen Liu, Charlotte Ehle, Aishwarya Iyer-Bierhoff, Bing Liu, Thorsten Heinzel, Shaojun Xing

**Affiliations:** 1 School of Basic Medical Sciences, Shenzhen University, Shenzhen 518055, China; 2 Center for Molecular Biomedicine, Institute of Biochemistry and Biophysics, Friedrich Schiller University Jena, 07745 Jena, Germany; 3 The First Affiliated Hospital of Shenzhen University, Shenzhen University, Shenzhen 518055, China

**Keywords:** sepsis, glycolysis, liver, SAHA, macrophage

## Abstract

Sepsis is a life-threatening medical emergency triggered by excessive inflammation in response to an infection. High mortality rates and limited therapeutic options pose significant challenges in sepsis treatment. Histone deacetylase inhibitors (HDACi), such as suberoylanilide hydroxamic acid (SAHA), have been proposed as potent anti-inflammatory agents for treating inflammatory diseases. However, the underlying mechanisms of sepsis treatment remain poorly understood. In this study, we investigated the effects of SAHA treatment in the lipopolysaccharide (LPS)-induced endotoxemia mouse model as it closely mimics the early stages of the systemic inflammation of sepsis. Our results demonstrate a reduced inflammatory mediator secretion and improved survival rates in mice. Using quantitative acetylomics, we found that SAHA administration increases the acetylation of lactate dehydrogenase (LDHA), and consequently inhibits LDHA activity. Notably, the reduced enzyme activity of LDHA results in a reduced rate of glycolysis. Furthermore, our experiments with bone marrow-derived macrophages (BMDMs) show that SAHA administration reduced oxidative stress and extracellular ATP concentrations, ultimately blunting inflammasome activation. Overall, our study provides insights into the mechanism underlying SAHA’s therapeutic effects in sepsis treatment and highlights LDHA as a potential target for developing novel sepsis treatment.

## 1. Introduction

Sepsis is a severe medical condition characterized by an excessive immune reaction and organ dysfunction of the host resulting from dysregulated host response to an infection [1]. It is responsible for approximately 11 million casualties annually, accounting for 20% of global mortality [2]. Despite decades of intensive research, the lack of specific clinical symptoms and the absence of effective drugs are still major therapeutic challenges in sepsis treatment [3,4,5,6,7] while improper or untimely treatment can rapidly lead to the massive release of inflammatory mediators, ultimately leading to multiorgan failure and even death [8]. Therefore, elucidating the mechanisms underlying the immune response of sepsis is critical to developing novel treatment options. 

The liver plays a critical role in sepsis as it is mainly responsible for regulating key metabolic, homeostatic, and host defense activity [9]. Considering the critical role of the liver in sepsis, the injury of the liver is a main event in the genesis and amplification of multiple organ failure which greatly contributes to the high mortality rate between 30%–50% in sepsis and septic shock [10,11]. Liver resident macrophages, commonly named Kupffer cells (KCs), are predominantly derived from fetal liver monocytes that constitute 80%–90% of the tissue macrophages present in the body. They exhibit a pronounced endocytic and phagocytic capacity to fight against pathogen invasion and constitute the major source of proinflammatory cytokines produced in the liver [12,13,14,15]. Growing evidence has revealed that the dysregulation of liver functions is frequently combined with the abnormal energy metabolism of KCs [16,17,18]. Under sepsis-induced hypoxia and oxidative stress, KCs largely depend on anaerobic glycolysis to meet their energy demands. Impaired glycolysis limits the supply of cellular ATP, resulting in defective phagocytosis, reactive oxygen species (ROS) production, and the secretion of inflammatory mediators [19,20,21,22]. Histone deacetylases (HDACs) are important players in the regulation of histone and non-histone protein acetylation. They exert their regulatory function by removing the acetyl group from acetyl-lysine residues, altering gene-expression patterns and affecting diverse aspects of protein function [23,24]. Deregulated HDAC activity is closely associated with the pathogenesis of cancer and inflammatory diseases [25]. Histone deacetylase inhibitors were discovered not only as anti-cancer drugs but also as potent anti-inflammatory agents. Suberoylanilide hydroxamic acid (SAHA), a class I HDACs (HDAC1, HDAC2 and HDAC3) and class II HDACs (HDAC6) inhibitor, was first approved by the U.S. Food and Drug Administration for T-cell lymphoma treatment [26,27]. Later, SAHA was applied as an immune modulator in a mouse sepsis model. SAHA treatment of sepsis has been shown to effectively reduce the secretion of inflammatory cytokines, such as TNF-α and IL-6, by downregulating the MyD88-dependent pathway. Additionally, SAHA treatment suppresses the activation of coagulation cascades, leading to the attenuation of coagulation protein and platelet consumption [25,28]. Still, the underlying mechanism of how SAHA restricts the inflammatory response in sepsis via transcription and post-translational modifications remains to be elucidated.

The present study demonstrates that SAHA increases survival rates in an LPS-induced endotoxemia mouse model, which mimics the initial systemic stage of sepsis. Our results reveal that SAHA achieves its therapeutic effect by suppressing inflammatory mediator secretion through the attenuation of the anaerobic glycolysis pathway, Taken together, our results suggest that SAHA-mediated targeting of the glycolysis pathway is a potent approach to treat LPS-induced sepsis in mice.

## 2. Results

### 2.1. SAHA Inhibits Liver Inflammation and Reduces Mortality in the LPS-Induced Sepsis Mouse Model

**Figure 1:**
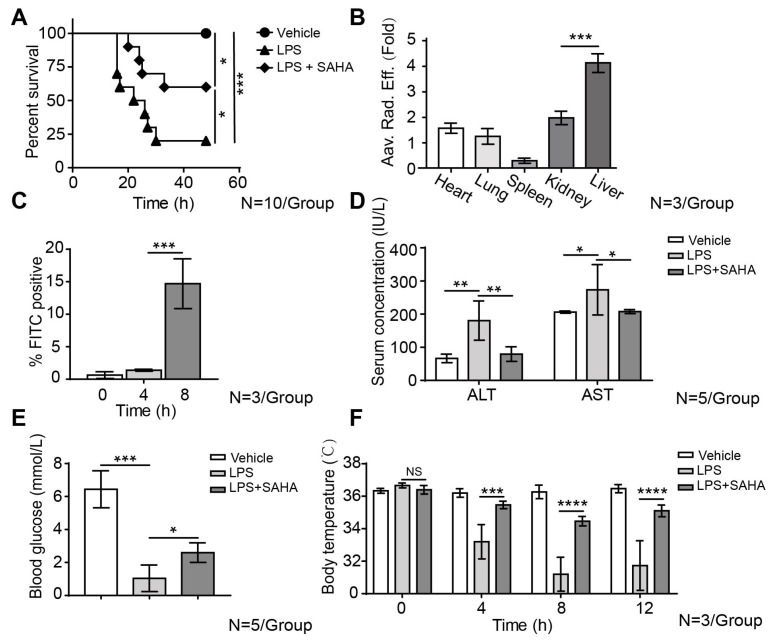
SAHA treatment reduces tissue damage and mortality in mice with LPS-induced sepsis. (**A**) Kaplan–Meier survival analysis comparing the LPS group to the LPS plus SAHA group in C57BL/6 mice injected with SAHA (50 mg/kg i.p.) or DMSO 1 h after LPS injection (30 mg/kg i.p.) (*N* = 10 animals/group). (**B**) In vivo animal imaging showing the biodistribution of FITC-conjugated LPS (1 mg/kg) in different organs 8 h after i.p. injection. Fold changes were calculated based on radiation efficacy divided by organ volume to indicate the distribution of LPS (*N* = 3 animals/group). (**C**) Flow cytometry analysis of FITC-labeled LPS (1 mg/kg i.p.) in the liver post i.p. injection on 4 and 8 h (*N* = 3 animals/group). (**D**,**E**) Serum alanine aminotransferase (ALT), aspartate transaminase (AST), and blood glucose levels were determined by auto-biochemical analyzer profiling (*N*= 5 animals/group). (**F**) Core body temperature changes of the control group, LPS groups and LPS plus SAHA group at indicated time points (*N* = 3 animals/group). Results in (**A**–**F**) are shown as averages ± SD. * *p* < 0.05; ** *p* < 0.01; *** *p* < 0.001; **** *p* < 0.0001, by one-way ANOVA.

To mimic the symptoms of sepsis patients in an animal model and to evaluate the in vivo therapeutical effects of SAHA in sepsis, we examined the effect of SAHA treatment in the mouse sepsis model induced via the intraperitoneal (i.p.) injection of LPS. Mice were segregated into three groups (*n* = 10/group): a vehicle group, an LPS (30 mg/kg) treatment group, and an LPS plus SAHA (50 mg/kg) treatment group. During the acute phase of sepsis, the excessive secretion of inflammatory mediators triggers an overwhelming immune response that results in the uncontrolled depletion of peripheral immune cells. Our study revealed a significant depletion of white blood cells in the peripheral blood, including lymphocytes and granulocytes, in septic mice (lymphopenia) which was ameliorated by SAHA treatment (Figure S1). The mortality rate in the LPS treatment group was observed to be 80%. In contrast, the LPS plus SAHA treatment group exhibited a mortality rate of 40%, representing a 50% decrease compared to the LPS treatment group (Figure 1A). LPS is a prototypical example of pathogen-associated molecular patterns (PAMPs) released by Gram-negative bacteria and is a major contributor to mortality in sepsis. We then asked the question of which internal organ is most vulnerable after LPS administration. To determine the concentration asymmetry of LPS among different organs, green-fluorescent Alexa Fluor 488 conjugated LPS was injected intraperitoneally. In vivo, fluorescence imaging revealed that the liver section exhibited a significantly higher concentration of LPS compared to other organs based on fluorescence intensity (Figure 1B and Figure S2). This result was further validated by flow cytometry analysis on fresh liver samples isolated from mice after FITC-LPS injection at indicated time points. The proportion of FITC-positive cells began to increase after 4 h post-injection, accumulating to substantial levels at 8 h after injection (Figure 1C). Therefore, we chose to conduct a further in vivo experiment at 8 h post-LPS injection, aiming to understand whether SAHA has the potential to mitigate the deleterious effects of LPS on the liver. To determine whether the accumulated LPS led to liver injury, we measured the levels of serum aspartate aminotransferase (AST) and alanine aminotransferase (ALT) which are widely recognized as important indicators of liver damage. SAHA treatment reduced AST and ALT levels, indicating that it ameliorated LPS-induced liver damage (Figure 1D).

The diagnostic assessment of liver tissue primarily relies on examining liver sections with Hematoxylin-Eosin (HE) staining, as shown in Figure S3. In vehicle-treated mice, healthy hepatic cells were observed a with well-defined cytoplasm and nucleus, as well as a well-brought-out central vein. However, in the other two treatment groups, there are indications of liver damage at various levels. Livers isolated from the LPS and SAHA co-treated group showed the reduced infiltration of mononuclear cells, less centrilobular necrosis, decreased congestion in the sinusoids, and minute foci of hepatic cell necrosis as compared to the LPS group.

A critical role of the liver is regulating glucose homeostasis and impairment of liver function may lead to metabolic abnormalities, including a reduction in gluconeogenesis. In sepsis, hypoglycemia is associated with poor outcomes and increased mortality in the clinic. In our study, we observed a significant decrease in blood glucose levels in mice treated with LPS while the administration of SAHA ameliorates hypoglycemia (Figure 1E). Additionally, we observed that LPS treatment induced hypothermia in mice, which is detrimental considering the finding that septic patients who present hypothermia have higher mortality rates than febrile patients. Interestingly, the administration of SAHA appeared to have a significant effect on maintaining the homeostasis of core body temperature in mice (Figure 1F). Together, our findings suggest that the administration of SAHA exerts a beneficial effect in septic mice, as it increased survival rates and reduced liver damage.

### 2.2. SAHA Suppresses Genes Involved in LPS-Induced Activation of the Glycolysis Pathway

**Figure 2:**
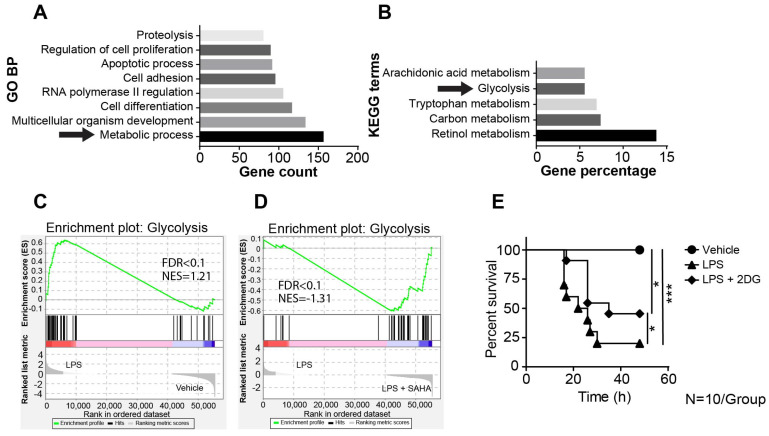
SAHA suppressed glycolysis through transcriptional regulation. (**A**,**B**) Mice were treated with LPS (30 mg/kg i.p.) and SAHA (50 mg/kg i.p.), and after 8 h, the liver was harvested for bulk−RNA sequencing. Differentiated expressed genes above 1-fold were listed, and gene ontology enrichment and KEGG pathway analysis were performed (*N* = 3 animals/group). (**C**,**D**) Gene expression was ordered according to their ranked ratios, and gene set enrichment analysis (GSEA) of the glycolysis pathway was performed to compare the vehicle-treated group with the LPS-treated (30 mg/kg i.p.) group (**C**), and the LPS-treated (30 mg/kg i.p.) group with the LPS (30 mg/kg i.p.)-plus SAHA-treated (50 mg/kg i.p.) group (**D**) (*N* = 3 animals/group). (**E**) Kaplan–Meier survival analysis comparing the LPS (30 mg/kg i.p.) group to the LPS (30 mg/kg i.p.) plus SAHA (50 mg/kg i.p.) group in C57BL/6 mice injected with 2DG (100 mg/kg i.p.) 1 h after LPS injection (30 mg/kg i.p.) (*N* = 10 animals/group). Results in (**E**) are shown as averages ±SD. * *p* < 0.05; *** *p* < 0.001, by one-way ANOVA.

To investigate mechanisms underlying SAHA-dependent protective effects on LPS-induced sepsis, we isolated the liver from vehicle treated, LPS treated and LPS plus SAHA-treated mice in each group (*n* = 3 per group), and the transcriptomes were analyzed by RNA Sequencing (RNA-Seq). We utilized the DESeq2 algorithm to select the differentially expressed genes (DEGs). We identified 3529 variable genes in the vehicle treated group compared to the LPS treated group and identified 222 variable genes in the LPS-treated group compared to the LPS-plus-SAHA-treated group with an adjusted *p*-value < 0.01 and an absolute value of log2-fold change ≥ 1. Previous studies have shown that a systematic activation of the immune system by LPS stimulation contributed to the severe inflammatory response characterized by an excessive release of cytokines and chemokines. The results obtained through RNA-Seq demonstrated a significant shift of the gene expression profile towards a pro-inflammatory state following the administration of LPS. This is evident from the upregulation of liver-specific acute phase protein-coding genes, such as *Lcn2*, *Saa1* and *Mt1*, indicated by the volcano plot (Figure S4). To further refine the responsive pathway, GO enrichment analysis was conducted to obtain GO terms of significant DEG enrichment using the DAVID database. The top eight significant biological processes were shown in the LPS-treated versus LPS-plus-SAHA-treated group, among which were apoptotic, cell differentiation, and adhesion processes that are known to be associated with sepsis pathogenesis. Notably, metabolic process genes emerged as the most prominent ontology variable, indicating the potential involvement of metabolic processes in sepsis pathogenesis (Figure 2A).

Subsequently, we focused on the investigation of the pathway level to pinpoint the specific metabolic pathway targeted by SAHA. To this end, the KEGG pathway enrichment analysis was applied to DEGs among metabolic-related genes identified in the GO analysis. This analysis revealed five predominant enriched metabolic pathways (*p*-value < 0.01) (Figure 2B). To elucidate the effects of SAHA on the glycolysis pathway, we applied the gene set enrichment analysis (GSEA) using the hallmark genes from the Molecular Signature Database (MSigDB). Among investigated pathways, LPS exclusively upregulated the anaerobic glycolysis pathway (Figure 2C), which was subsequently downregulated by SAHA treatment (Figure 2D). In contrast, the other pathways were found to be suppressed by LPS, and SAHA treatment further reduced their activation (Figure S5), suggesting that SAHA exerts its therapeutic effects independently of those pathways. Moreover, published findings suggest that the exaggerated inflammatory response relies on anaerobic glycolysis to meet its energy demands. The increased metabolic demand activates hypoxia signaling (Figure S6), alters the cellular energetics and triggers the shift towards glycolysis and reduced oxygen consumption. This is consistent with the finding from our enrichment analysis, which indicates that SAHA exerts its anti-inflammatory effects by regulating the anaerobic glycolysis in the sepsis model. To determine whether this suppressed glycolysis has a positive impact on the survival rate of septic mice, the potent GLUT1 inhibitor 2-Deoxy-d-Glucose (2DG) was injected intraperitoneally with a dosage of 100 mg/kg body weight one hour after LPS administration to inhibit glycolysis in septic mice. The inhibitor 2DG greatly increased the survival rate in septic mice, as indicated in Figure 2E. These results suggest that SAHA administration suppressed the glycolysis pathway and that this suppression results in an increased survival rate in septic mice.

### 2.3. The Acetylation Pattern of Glycolytic Enzymes Is Altered in Septic Mice

**Figure 3:**
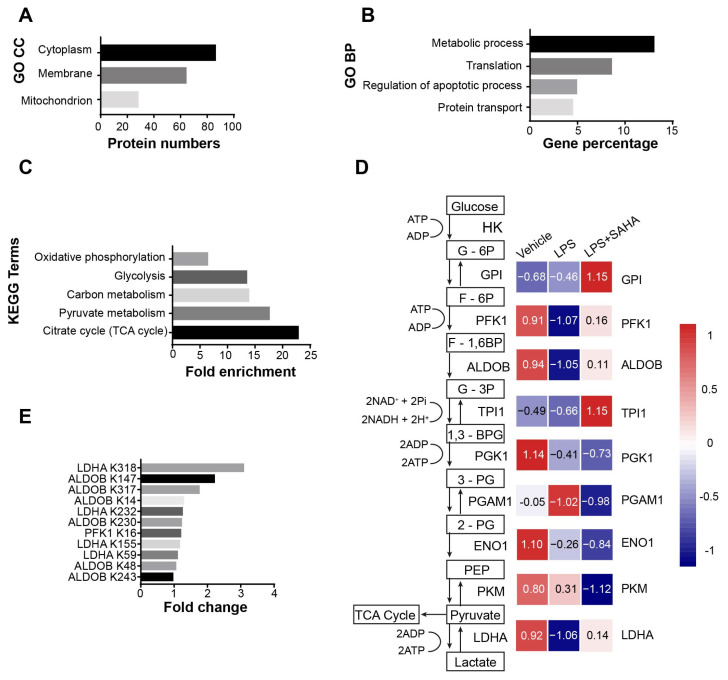
SAHA suppresses the activation of the glycolysis pathway after LPS administration. (**A**) Mice were treated as indicated above, and after 8 h, the liver was harvested and subjected to protein acetylation sequencing. Changes in acetylation levels above 0.5-folds in LPS (30 mg/kg i.p.) compared to LPS (30 mg/kg i.p.)-plus-SAHA (50 mg/kg i.p.) group were subjected to ontology functional classification of cellular component analysis (*N* = 3 animals/group). (**B**) Cytoplasmic proteins above were subjected to functional enrichment analysis to show the top-ranked biological processes in the LPS treated (30 mg/kg i.p.) group compared to the LPS (30 mg/kg i.p.)-plus SAHA-treated (50 mg/kg i.p.) group (*N* = 3 animals/group). (**C**) Genes involved in metabolic processes were subjected to KEGG pathway analysis, and the top-ranked pathways are shown (*N* = 3 animals/group). (**D**) Heatmap showing the acetylation levels of glycolytic enzymes in the indicated treatment condition (*N* = 3 animals/group). (**E**) In the LPS (30 mg/kg i.p.) versus LPS (30 mg/kg i.p.)-plus-SAHA (50 mg/kg i.p.) treatment condition, the acetylation sites of LDHA, ALDOB, and PFK1, along with their acetylation fold changes are shown (*N* = 3 animals/group).

SAHA is a potent inhibitor of class I and class II histone deacetylases, which inhibits the removal of the acetyl group on lysine residues on both histone and non-histone proteins. We investigated whether SAHA could regulate the immune response in sepsis through its HDAC inhibitor activity. Hence, we determined whether SAHA orchestrates the activation status of the glycolysis pathway by regulating acetylation levels on glycolytic enzymes. To verify this hypothesis, antibody enrichment-based label-free quantitative acetylome analysis was performed on the liver samples isolated from mice treated with vehicle, LPS and LPS plus SAHA. A total of 1085 acetylation sites across 764 proteins were detected in the LPS- versus vehicle-treated group. In the LPS-plus-SAHA versus LPS group, we identified 843 acetylation sites among 656 proteins. In addition to increased global histone acetylation levels (Figure S7), SAHA also substantially influenced the acetylation status of non-histone proteins. Interestingly, GO analysis of the cellular component term in the LPS plus SAHA treatment group compared with the LPS group revealed that most SAHA-regulated acetylated proteins are located in the cytoplasm (Figure 3A), suggesting that SAHA may exert its effects also by regulating non-histone protein acetylation. This observation is supported by a significant increase in the number of upregulated acetylation sites following LPS plus SAHA treatment, as compared to the LPS treatment group (Figure S8). Moreover, to elucidate how SAHA exerts its acetylation regulatory effects towards non-histone proteins, GO enrichment analysis was applied on proteins having differentially regulated acetylation sites (adjusted *p*-value < 0.01 and absolute value of fold change > 0.5) in the LPS versus vehicle (Figure S9) and LPS-plus-SAHA versus LPS treatment (Figure 3B). As the acetylation-enrichment analysis in LPS-plus-SAHA compared to the LPS treatment group pinpointed the metabolic process as the most significantly regulated biological process, a follow-up KEGG pathway enrichment analysis was applied to elucidate which metabolic pathway could be a predominant target of regulation via acetylation. The five top significant pathways are shown in the LPS-treated versus LPS-plus-SAHA-treated group (Figure 3C).

The dramatic upregulation of glycolysis, but not other pathways, was observed following LPS treatment and subsequently decreased with SAHA treatment (Figures S5 and S10). We showed that inhibiting glycolysis suppresses the secretion of inflammatory mediators IL6 and TNF alpha, leading to increased survival rates in septic mice (Figure 2E and Figure S13). To exclude indirect acetylation effects such as feedback mechanisms and compensatory pathways after SAHA treatment, we only focused on enzymes, which were downregulated in their acetylation after LPS treatment and restored after SAHA for further analysis. A heat map was generated to depict the acetylation levels of glycolytic enzymes in different treatment conditions (Figure 3D). The results revealed that three glycolytic enzymes including lactate dehydrogenase (LDHA), phosphofructokinase (PFK) and aldolase (ALDOB) met the criteria. This suggests that SAHA, through its HDAC inhibitory function, may inhibit glycolytic enzyme activity and regulate the glycolysis pathway by increasing the acetylation levels of crucial glycolytic enzymes. In Figure 3E, acetylation levels on each lysine site are shown in the LPS versus LPS plus SAHA treatment group. Taken together, acetylomic analysis revealed that the effects of SAHA are largely dependent on regulating non-histone proteins. The suppression effects on the glycolysis pathway are linked to the acetylation changes of glycolytic enzymes.

### 2.4. SAHA Attenuates Glycolysis via LDHA Acetylation on the Lysine 318 Site

**Figure 4:**
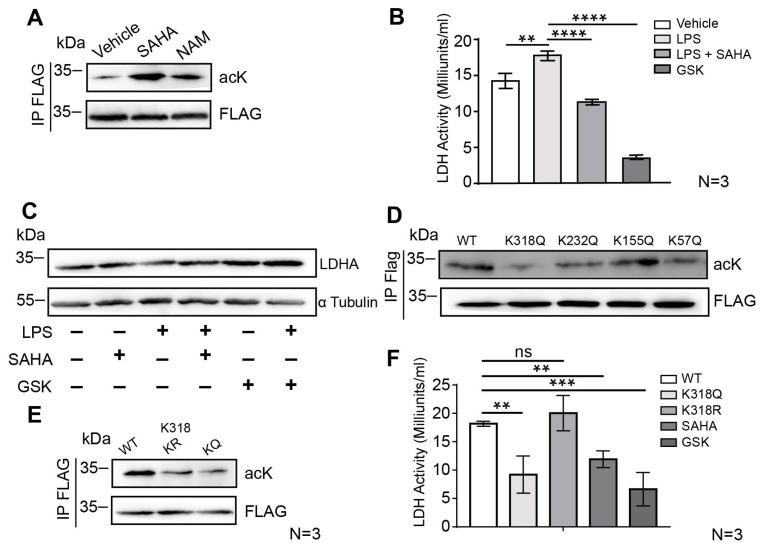
Acetylation at K318 suppresses the enzymatic activity of LDHA. (**A**) HEK293T cells were transfected with FLAG-tagged LDHA plasmid, and Flag-LDHA was immunoprecipitated after treatment. The acetylation levels of LDHA were detected by the pan-acetylation antibody. (**B**) BMDMs were treated with LPS (100 ng/mL), SAHA (1 μM) and LDHA inhibitor GSK (1 μM), and the enzyme activity of LDHA was determined. (**C**) BMDMs were treated as indicated, and the levels of LDHA protein were determined. (**D**) HEK293T cells were transfected with point-mutated LDHA plasmid (K318, K232, K155, and K57), and Flag-LDHA was immunoprecipitated. The acetylation levels of LDHA were detected by the pan-acetylation antibody. (**E**) The acetylation level of point-mutated LDHA protein (KQ and KR mutation) at the lysine 318 site was determined. (**F**) The enzyme activity of point-mutated LDHA at lysine K318 was determined. Results in (**B**,**F**) are shown as averages ± SD. ** *p* < 0.01; *** *p* < 0.001; **** *p* < 0.0001, by one-way ANOVA.

Our results indicate that PFK, ALDOB, and LDHA, among other glycolytic enzymes, exhibit reduced acetylation levels in the LPS-treatment group, which are subsequently preserved upon concomitant SAHA treatment. Considering the crucial role of LDHA in sustaining anaerobic glycolysis, LDHA deficiency has been associated with impaired glucose metabolism. Previous research reported that enzyme activity and protein stability of LDHA is known to be regulated by various post-translational modifications. As shown in Figure 3E, lysine 318 (K318) of LDHA exhibits the highest acetylation fold change among all other sites examined. Therefore, we investigated whether upregulating LDHA K318 acetylation plays a role in regulating the enzyme activity of LDHA. To test this hypothesis, we first determined whether SAHA-dependent HDACi activity augments LDHA acetylation levels. We transiently expressed Flag-tagged LDHA in HEK293T cells that were treated with vehicle, SAHA or the sirtuin inhibitor, nicotinamide (NAM), and the acetylation of immunoprecipitated LDHA was detected by pan-acetyl lysine antibody using Western blot. SAHA but not NAM greatly enhanced the acetylation of LDHA, implying that LDHA acetylation is mainly regulated by class I/II/IV HDACs but not class III (Figure 4A).

To assess SAHA’s effect on LDHA activity, we measured LDHA activity in BMDMs treated with LPS or LPS plus SAHA. Mouse BMDMs were subjected to either LPS or LPS plus SAHA treatment for 6 h. As shown in Figure 4B, LDHA activity was enhanced upon LPS treatment, whereas SAHA treatment significantly diminished the activity of LDHA, LDHA inhibitor GSK2837808A (GSK) served as a positive control (Figure 4B). To exclude that this suppression is due to effects on transcriptional and translational regulation, the expression levels of mRNA and protein of LDHA after SAHA treatment were examined, respectively. We performed Western blot assay to detect the protein level of LDHA after LPS and SAHA treatment. Since SAHA neither altered the mRNA expression of LDHA nor the LDHA protein level (Figure 4C and Figure S11), the decreased enzyme activity was most likely caused by post-translational modification rather than transcriptional or translational regulation. To further explore how acetylation regulates LDHA enzyme activity, we mutated each of four putative acetylation sites from acetylome profiling data including lysines K57, K155, K232 and K318 individually to glutamine (Q) to mimic an acetylated lysine state, and examined the changes in acetylation levels of LDHA. Both K57 and K318 mutations result in a reduction in LDHA acetylation (Figure 4D). To test the effect of K318 acetylation, we mutated K318 from lysine to arginine (R) to mimic an unacetylated lysine state (Figure 4E), then compared the activity of LDHA K318R and LDHA K318Q mutants to that of wild-type LDHA (Figure 2F). According to the enzyme activity assay, LDHA K318Q displayed approximately 50% of the wild-type activity, while the LDHA K57Q mutation does not affect LDHA enzyme activity. Together, these results demonstrated that the acetylation of LDHA at K318, an evolutionarily conserved site in mammals (Figure S12), inhibits LDHA activity.

### 2.5. SAHA Blunts Macrophage Mobilization and Inflammasome Activation

**Figure 5:**
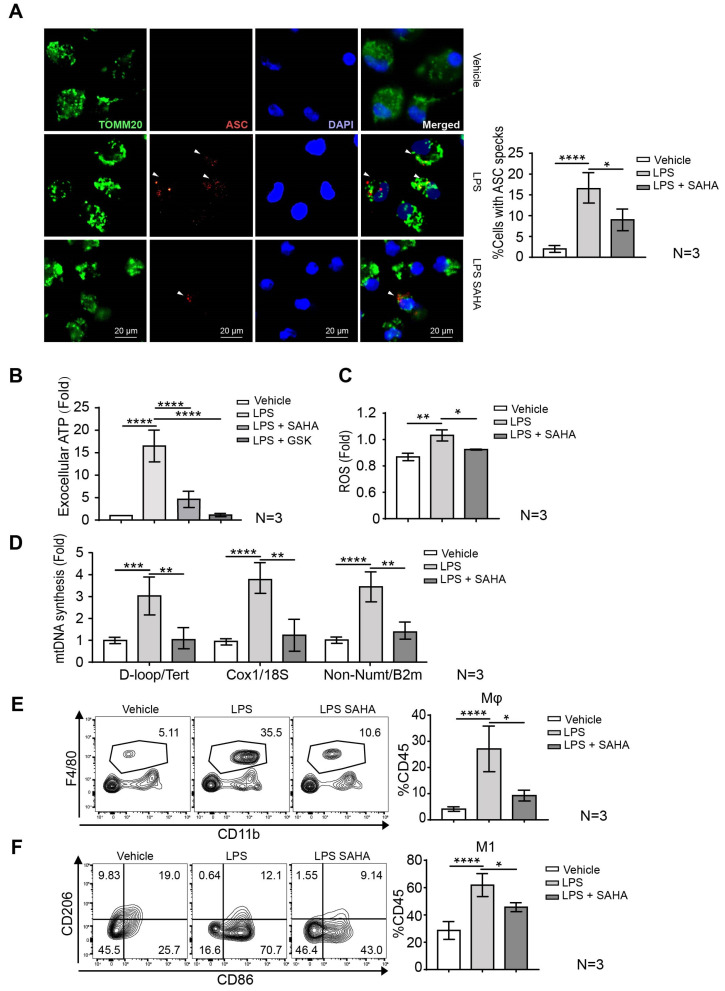
SAHA inhibits LPS-induced macrophage mobilization and inflammasome activation. (**A**) Representative fluorescent microscopy images of BMDMs co-stained for TOMM20 and ASC before and after LPS (100 ng/mL) priming followed by SAHA (1 μM) treatment. DAPI stains nuclei. Arrows indicate ASC specks. Scale bars, 100 μm (*n* = 3). Percentages of cells shown in (**A**) with ASC specks. *n* = 100 cells per group from 3 independent experiments, magnification ×100. (**B**) Extracellular ATP measurement of SAHA and GSK (1 μM) treatment on LPS (100 ng/mL)-primed BMDMs, analyzed by ATP-based luminescence in absolute values. (**C**) Relative ROS amounts at the indicated time point were detected by the chemiluminescent probe in BMDMs. (**D**) Relative mtDNA amounts in LPS-primed (100 ng/mL) BMDMs with or without SAHA (1 μM) treatment. The ratio of mitochondrial DNA to nuclear DNA was shown by gene pairs of D-loop(mt)/Tert(n), Cox1(mt)/18S(n), and not inserted into nuclear DNA/B2m(n), respectively. (**E**) The proportion of KCs was determined by flow cytometry. (**F**) The proportion of M1 macrophages was determined based on CD86 expression. Results in (**A**–**F**) are shown as averages ± SD. * *p* < 0.05; ** *p* < 0.01; *** *p* < 0.001; **** *p* < 0.0001, by one-way ANOVA.

Macrophages undergo pyroptotic cell death via inflammasome activation after LPS stimulation. We have detected increased cytokine secretion levels and FITC signal in F4/80 and CD11b double-positive KCs in mouse livers after FITC-conjugated LPS administration (Figures S13 and S14). Previous evidence shows that pyroptosis is one important cause of multiple organ failure in sepsis. In addition, the activation and mobilization of macrophages largely depend on glycolysis to fuel their energy consumption. Therefore, we next investigated whether this suppression of glycolysis by SAHA affects pyroptosis in macrophages. As the assembly of apoptosis-associated speck-like protein (ASC) is the hallmark of inflammasome activation to initiate pyroptotic cell death, we monitored the assembly of ASC by fluorescence microscopy in LPS-primed BMDMs with or without SAHA treatment. SAHA reduced the number of ASC specks in LPS-primed BMDMs (Figure 5A). In addition, pyroptotic cells release cytosolic contents into the extracellular space including ATP (or extracellular ATP), a component of the well-described damage-associated molecular patterns (DAMPs). The recognition of extracellular ATP (eATP) by the P2 × 7 receptor, an ATP-sensing ion channel, initiates a series of events that culminate in the assembly of the NLRP3 inflammasome. We next addressed whether SAHA treatment affects eATP concentration in LPS-primed BMDMs (Figure 5B). The significantly elevated eATP concentration caused by LPS treatment was blunted after the administration of SAHA, and the LDHA inhibitor treatment group also showed a strong reduction in eATP. Moreover, the mitochondria in LPS-primed macrophages produce significantly higher levels of ROS in combination with newly synthesized mtDNA. Accordingly, we observed an increased synthesis of mtDNA and ROS production in LPS-primed BMDMs. The increased mtDNA and ROS production has previously been reported to induce the formation of oxidized mtDNA and the activation of the inflammasome. SAHA administration blunted the formation of oxidized mtDNA via suppressing ROS production as well as reduced mtDNA synthesis (Figure 5C,D). In addition, we assessed the proportion of macrophages among total CD45^+^ liver resident immune cells from the perfused fresh liver isolated from the vehicle, LPS and LPS plus SAHA group (Figure 5E), indicated by the notably higher CD11b and F4/80 double-positive cells in the LPS-treated group compared to the vehicle group. However, SAHA treatment blunted the mobilization of macrophages to the liver, as indicated by the reduced percentage of macrophages among total CD45^+^ cells. Similarly, LPS-induced macrophage polarization towards catabolic-dependent M1 status was also suppressed by SAHA treatment (Figure 5F). The treatment of SAHA exerts a universal influence on various immune cell populations within the liver. In septic mice, liver resident immune cells are highly susceptible to LPS-induced depletion as a consequence of persistent overactivation. SAHA has shown promising effects in suppressing the activation of T cells determined by its activation markers CD25 and CD69 (Figure S15). Taken together, our findings suggest that SAHA has significant inhibitory effects on inflammasome activation, further preventing the activation of macrophage towards pro-inflammatory status.

## 3. Discussion

Our study reveals that SAHA is capable of suppressing the excessively activated inflammation in septic mice by downregulating critical inflammatory mediators and enhancing survival rates via regulation of the glycolysis pathway. Under the activation of anaerobic glycolysis, the key function of LDHA is to convert pyruvate to lactate, thus converting NADH into NAD^+^ to maintain the activated glycolysis. The reduction in the catalytic activity of LDHA will subsequently impair glycolysis by limiting the supply of NAD^+^, thus limiting the Warburg effect and exerting anti-inflammatory functions [29]. We have shown that SAHA suppresses the enzyme activity of LDHA by maintaining a hyperacetylated state of LDHA. This in turn resulted in a great reduction in the enzymatic activity of hyperacetylated LDHA. The decline in LDHA activity due to SAHA treatment exhibited a correlation with the enzyme activity findings derived from the ectopic expression of LDHA carrying a lysine to a glutamine point mutation at position K318. While a previous study demonstrated that the lysine-5 acetylation of LDHA leads to negative regulation of its activity and subsequent reductions in cell proliferation and migration, the effects of decreased LDHA enzyme activity in inflammation response signalling have not yet been investigated [30].

In sepsis disease, the substantial activation of the glycolysis pathway is considered a hallmark of an excessive inflammation response after pathogen stimulation which leads to destructive disease states [31]. In recent years, mounting evidence has highlighted the interplay between inflammation and metabolic reprogramming in immune cells [32]. Given that glycolysis is a key process to generate ATP and biosynthetic precursors to fuel cellular reactions during inflammatory disease progression, targeting the glycolysis process represents a promising therapeutic strategy for inflammatory diseases. The ability of HDACi to modulate the acetylation of both histone and non-histone proteins has long been considered a potent anti-inflammatory mechanism [33]. Although the therapeutic benefits of HDAC inhibitors in cancer and inflammatory diseases have been associated with various metabolic processes [34], the relationship between the acetylation of glycolytic enzymes and its effects on innate immune cells in sepsis remains poorly understood. Previous studies have indicated that SAHA confers beneficial effects in mice with sepsis induced by LPS injection or cecal ligation and puncture [25,35,36,37,38]. By harnessing the power of multi-omics studies in conjunction with various biochemical assays, we uncovered that the increased acetylation of glycolytic protein by SAHA acts as a barrier to limit the activation of glycolysis signaling.

In sepsis, the excessive immune response, particularly the secretion of cytokines by macrophages, plays a critical role in inflammatory-induced cell death. This process involves nuclear condensation, DNA fragmentation, and activation of the inflammasome, resulting in cell swelling and rupture that destroys cell membrane integrity and causes inflammatory necrosis. Previous research has demonstrated that eATP amplifies cellular oxidative stress, leading to increased ROS production and elevated mtDNA expression, which accelerates Ox-mtDNA production [39]. Subsequently, the inflammasome is activated after Ox-mtDNA enters the cytoplasm [40]. Our study shows that SAHA effectively inhibits the glycolysis pathway, thereby leading to a significant decrease in glycolysis-mediated ATP production. Moreover, SAHA treatment decreased ROS generation and mtDNA replication in LPS-primed BMDMs. We have also confirmed SAHA has an extensive ability to suppress inflammasome activation in BMDMs by fluorescent staining to indicate its anti-inflammatory effects. Although this study leverages the insights gained from the LPS-induced endotoxemia model in sepsis treatment, it is crucial to acknowledge that sepsis is a complex disease with three distinct stages including the initial systemic inflammation stage, immunosuppression stage and long-lasting immune disturbances stage. The endotoxemia model only mimics the initial inflammatory stage, and further research is warranted to evaluate the efficacy of SAHA in other sepsis models that recapitulate the various stages observed in real clinical settings.

## 4. Materials and Methods

### 4.1. Septic Model and SAHA Treatment in Mice

We used 6-8 week female C57BL/6 mice purchased from Guangdong medical laboratory animal center (Guangzhou, China). All animals were housed in a temperature-controlled room under a 12 h light/12 h dark cycle and pathogen-free conditions. The sepsis model was induced by an LPS (Sigma-Aldrich, St. Louis, MO, USA) intraperitoneal injection reported previously. Animals were randomly assigned to the following three groups (*n* = 10/group). (1) Vehicle-treated group (Control). (2) LPS-treated group (LPS): animals received LPS (30 mg/kg). (3) HDAC inhibitor-treated group (LPS plus SAHA), animals received Vorinostat (SAHA) (50 mg/kg) (MCE, Monmouth Junction, NJ, USA) in DMSO (MCE, Monmouth Junction, NJ, USA) 1 h after LPS (30 mg/kg) injection. To measure liver enzymes, plasma was collected 8 h after LPS injection, and the plasma ALT, AST and glucose levels were measured by Chemray 800 (Rayto, Shenzhen, China). The core body temperature was measured by a rectal thermometer (Etienne, Shenzhen, China). All animals were housed and handled following protocols approved by the Committee on the Use of Live Animals in Teaching and Research of Shenzhen University.

### 4.2. Intrahepatic Immune Cell Isolation

Hepatic lymphocytes were isolated using established gradient methods. In brief, the animal was sacrificed with CO_2_ inhalation. The liver was perfused through the portal vein with Ca^2+^ and Mg^2+^ free Dulbecco’s phosphate-buffered saline (DPBS, Hyclone, Logan, UT, USA) containing 2 mM EDTA at 37 °C for 5 min. Subsequently, the liver was removed and cut into small pieces before enzyme digestion. Then, the liver digestion process was conducted by 50 mL of Dulbecco’s Modified Eagle Medium (DMEM, Cytiva, Marlborough, MA, USA) containing 0.025% type IV collagenase (Sigma-Aldrich, St. Louis, MO, USA) at 37 °C for 5 min followed by 70 μm nylon cell strainer filtration to exclude any liver tissue debris, and then suspended in the suspension medium (DMEM supplement with 10% heat-inactivated FBS). After centrifugation at 1000× *g* for 5 min, the pellet was washed twice in the suspension medium. Cells were suspended in 40% Percoll (Sigma-Aldrich, St. Louis, MO, USA) layered over 80% Percoll for density centrifugation at 2000 rpm for 30 min. Enriched cells were collected from the interphase. The collected cells were washed twice with suspension medium and resuspended in DPBS for further analysis.

### 4.3. Histological and Staining

Based on the previously described protocol [41], in short, mice were sacrificed 8 h after treatment. The liver was dissected and fixed in 4% paraformaldehyde overnight. Then, all samples were embedded in paraffin and cut into 3 μm sections using the microtome (Leica, Wetzlar, Germany). Standard HE staining using hematoxylin/eosin was applied. For image analysis of histological sections, Aperio ImageScope software (Leica, Wetzlar, Germany) was used (v12.3.3.7014).

### 4.4. Immunofluorescent Staining

Mouse BMDMs cells were seeded on eight-chambered coverslips (ThermoFisher, Waltham, MA, USA). After the treatment, cells were washed twice with PBS and fixed in 4% paraformaldehyde for 20 min and washed in PBS again. Blocking was performed with buffer containing 10% fetal bovine serum, 0.1% Triton X-100, and 0.05% sodium azide in PBS. The cells then were incubated with PE Anti-ASC (ThermoFisher, Waltham, MA, USA), FITC Anti-TOMM20 (ThermoFisher, Waltham, MA, USA) and DAPI (Abcam, New Haven, CT, USA) overnight. Finally, cells were washed twice in PBS and treated with VectaShield to preserve fluorescence (Vector Laboratories, Newark, CA, USA). We used Image J software (Bethesda, MA, USA) to subtract the background, set the threshold and select the region of interest.

### 4.5. LPS Accumulation Studies

The accumulation of LPS in the liver was studied by the intraperitoneal injection of Alexa Fluor 488 conjugated LPS (ThermoFisher, Waltham, MA, USA). Animals were sacrificed as previously described, and the in vivo multispectral fluorescence imaging was performed using the in vivo imaging system (IVIS, Tucson, AZ, USA) spectrum (PerkinElmer, Waltham, MA, USA) with living image software (V4.4) to detect fluorescent densities in indicated organs. Then, the liver was harvested and digested. The fluorescent density in cell suspension was analyzed using flow cytometry (Cytoflex, Beckman Coulter, Brea, CA, USA). Data were analyzed by FlowJo software (FlowJo V10.6, Ashland, OR, USA).

### 4.6. Liver Transcriptome Profiling

Fresh livers isolated from mice in each indicated group were stored in liquid nitrogen and subsequently RNA-Seq analysis was performed (Azenta, Burlington, MA, USA) according to the protocol provided by Azenta. In brief, the total RNA of each sample was extracted using TRIZOL Reagent (Invitrogen, Waltham, MA, USA). The total RNA of each sample was quantified and qualified by Agilent 2100 Bioanalyzer (Agilent Technologies, Santa Clara, CA, USA). A weight of 1 μg total RNA with the RIN value above 6.5 was used for the following library preparation. Then, libraries with different indices were multiplexed and loaded on an Illumina HiSeq instrument according to the manufacturer’s instructions (Illumina, San Diego, CA, USA). Sequencing data were processed with quality control to remove technical sequences, including adapters, polymerase chain reaction (PCR) primers, or fragments thereof, and a quality of bases lower than 20, pass filter data of fastq format were processed by Cutadapt (V1.9.1) to be high-quality clean data. Then, mapped with reference genome sequences and annotations via the software Hisat2 (v2.0.1), the mapped data were further converted from known *gff* annotation files and indexed properly. The DESeq2 (DESeq2 1.16.1) normalized fold-changes were then used to estimate different expression genes among different treatment groups.

### 4.7. Omics Data Analysis

GOSeq (v1.34.1) was used to identify Gene Ontology (GO) terms that annotate a list of enriched genes with a significant adjusted *p* value less than 0.05. The KEGG analysis was applied through the DAVID bioinformatics resources database (https://david.ncifcrf.gov/(accessed on 21 February 2023)). Each pathway with an adjusted *p* value less than 0.05 as a filter to obtain significantly enriched KEGG pathways. Gene set enrichment analysis (GSEA) was performed with the downloaded GSEA software GSEA.

### 4.8. LDH Assay

In brief, Flag-LDHA was ectopically expressed in HEK293T cells, immunoprecipitated and eluted. LDH activity was evaluated under the manufacturer’s protocol (Sigma Aldrich, St. Louis, MO, USA). The assay is based on the conversion of lactate to pyruvate, which generates NADH, resulting in a change in absorbance at 450 nm.

### 4.9. Site-Directed Mutagenesis

The generation of the point mutated LDH-A plasmids using a site-direct mutagenesis kit (ThermoFisher, Waltham, MA, USA) under the manufacturer’s protocol. In short, to create the KR and KQ mutants in LDHA using Flag-tagged pCMV-tag2 alpha plasmid.

### 4.10. Primary Cell Culture

This isolation and culture of female mouse bone marrow macrophages were performed as previously described [42]. In short, the tibias and femurs were removed from female mice, and the bone marrow cells were extracted and cultured in DMEM media supplemented with 30% L929 conditioned media as a source of Macrophage colony-stimulating factor (MCSF).

### 4.11. Expression of Recombinant Protein

HEK293T cell line (ATCC, Manassas, VA, USA) was maintained in complete DMEM supplemented with 10% FBS and 1% Penicillin-Streptomycin Solution at standard cell culture conditions. Transfection was performed in 10 cm dishes with 10 mL culture media via the CaCl_2_ method. In short, each well was transfected with a mixture of 10 μg LDHA expression pCMV-Tag2 alpha plasmid or 10 μg GFP-expression plasmid, combined with 10 μg empty pBluescript plasmid. Added to 100 μL 2.5 M CaCl_2_ and 1 mL 2× BBS BES-buffered saline (ThermoFisher, Waltham, MA, USA) to a final volume of 2 mL. After 8 h post-transfection, the cell medium was replaced with a fresh medium and the cells were incubated for another 36 h to check expression efficiency for GFP expression under the microscope before harvest.

### 4.12. qRT-PCR Analysis

qRT-PCR analysis was performed using the 7500 Real-Time PCR machine (Applied Biosystems, Waltham, MA, USA). Total RNA from the cell was isolated using the Trizol reagent (Invitrogen, Waltham, MA, USA). Reverse transcription was performed using 1 μg total RNA using a reverse transcription kit under the manufacturer’s protocol (ThermoFisher, Waltham, MA, USA). Amplification reactions were set up in 15 μL reaction volumes containing SYBR Green PCR Master Mix (Applied Biosystems, Waltham, MA, USA) and amplification primer sequences are listed in Table S1.

### 4.13. Immunoprecipitation and Western Blot Analysis

The immunoprecipitation assay was performed as previously described [43]. In short, HEK293T cells expressing epitope-tagged protein were lysed in AM-300 buffer, and lysates were collected and incubated with M2 anti-FLAG beads (Sigma-Aldrich, St. Louis, MO, USA) in 4 °C for 4 h. Then, the beads were spun down and washed in lysis buffer before the protein elution process by boiling at 95 °C in Laemmli buffer. Samples were run on 12% gels and transferred to hydrophilic polyvinylidene fluoride (PVDF) membranes. The membrane was blocked for 1 h at room temperature with 5% of milk solution in TBST buffer before incubating with primary antibody at 4 °C overnight. The membrane was washed in TBST buffer for 15 min before incubating with appropriate HRP-conjugated secondary antibody at room temperature for 1 h, then visualized with chemiluminescence reagents (Cyanagen Srl, Bologna, Italy).

### 4.14. LC-MS/MS Analysis

Fresh livers isolated from mice in each indicated group were stored in liquid nitrogen and subsequently performed the acetylome analysis (PTM-Biolabs, Hangzhou, China). The quantitative protein acetylation study was carried out by the acetylation protein enrichment method and followed by the high-resolution liquid chromatography–mass spectrometry LC-MS/MS. A downstream bioinformatics study of quantitated acetylome data was performed after normalization with protein quantification.

### 4.15. Flow Cytometry

Cells isolated from the liver as previously described were subjected to FACS analysis. Cells were dispelled into single cells. Cells were collected and incubated with BV510- Zombie Dye for 10 min at room temperature. For KC analysis, cells were incubated with FITC-conjugated, PE-conjugated, BV421-conjugated, APC-conjugated and PE-CY7-conjugated antibodies that recognized lineage markers, CD45, CD11b, F4/80, CD86 and CD206 for 20 min at 4 °C. For T cell and B cell analysis, cells were also firstly incubated with Zombie Dye and subsequently incubated with PE-conjugated, FITC- conjugated and APC- conjugated markers, CD45, CD3 and CD19 for 20 min at 4 °C. All antibodies are commercially available and used at the recommended concentration.

### 4.16. Reactive Oxygen Species Measurement

To measure reactive oxygen species, the luminescent probe L-012(Wako Chemical Corporation, Doshomachi Chuo-ku, Japan) was used. BMDMs were pre-seeded in 96-well plates and BMDMs culture medium containing 1mM L-012 was add in indicated treatment groups. Luminescence was recorded at indicated time points with the Tecan plate reader infinite 200 (Tecan, Männedorf, Switzerland).

## 5. Conclusions

Our study uncovered that hyperacetylation of LDHA led to a significant reduction in its enzyme activity, thereby attenuating the overactivation of the glycolytic pathway and consequently, crippling inflammasome activation. Moreover, by inhibiting the glycolysis pathway in septic mice, we observed a greatly reduced inflammatory mediator secretion and subsequently increased survival rate. The LPS-induced endotoxemia model closely mimics the early systemic inflammatory stages in septic patients, characterized by overwhelming inflammatory mediator secretion and liver dysfunction, which contributes to the observed mortality rate in clinical settings. Considering that SAHA has been approved by the FDA for the treatment of T-cell lymphoma [26], and due to its safety and low cost, further exploration is required in clinical settings to assess the therapeutic efficacy of the aforementioned findings.

## Data Availability

Data are contained within the article or Supplementary Materials. Raw data and materials are available from the authors upon reasonable request.

## Supplementary Materials

The following supporting information can be downloaded at: https://www.mdpi.com/article/10.3390/ijms241512448/s1.
