# Supplementary material for: Histone Deacetylase Inhibitor (SAHA) Reduces Mortality in an Endotoxemia Mouse Model by Suppressing Glycolysis
Source: Int J Mol Sci. 2023 Aug 4;24(15):12448. doi: 10.3390/ijms241512448 (PMC10418975; doi:10.3390/ijms241512448)
Supplement: Supplementary file 1 [file ijms-24-12448-s001.zip › ijms-2527012-supplementary.pdf]

Supplementary Materials

Figure S1

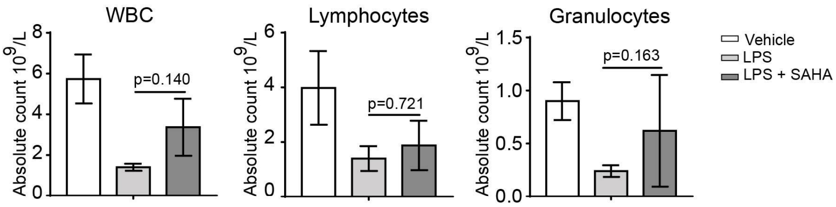

Figure S2

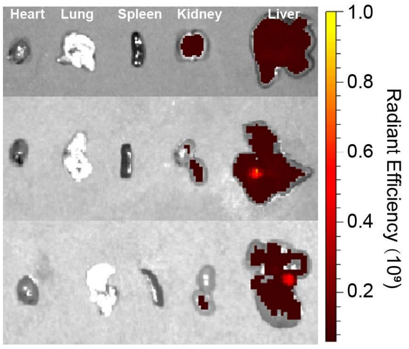

Figure S3

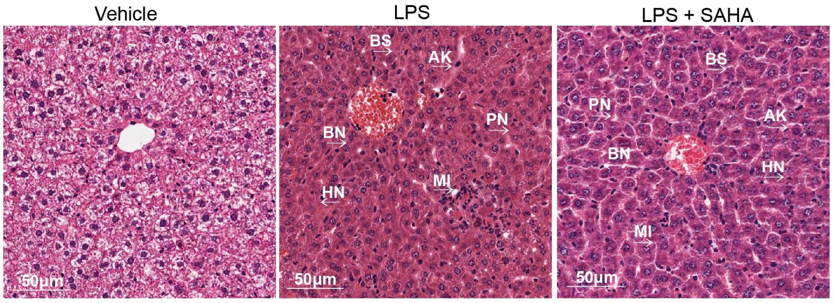

Figure S4

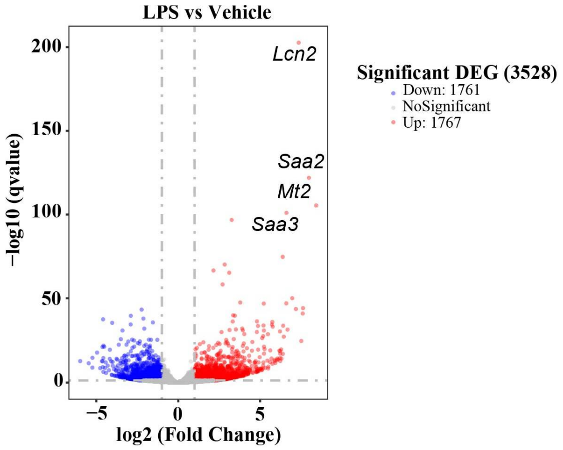

Figure S5

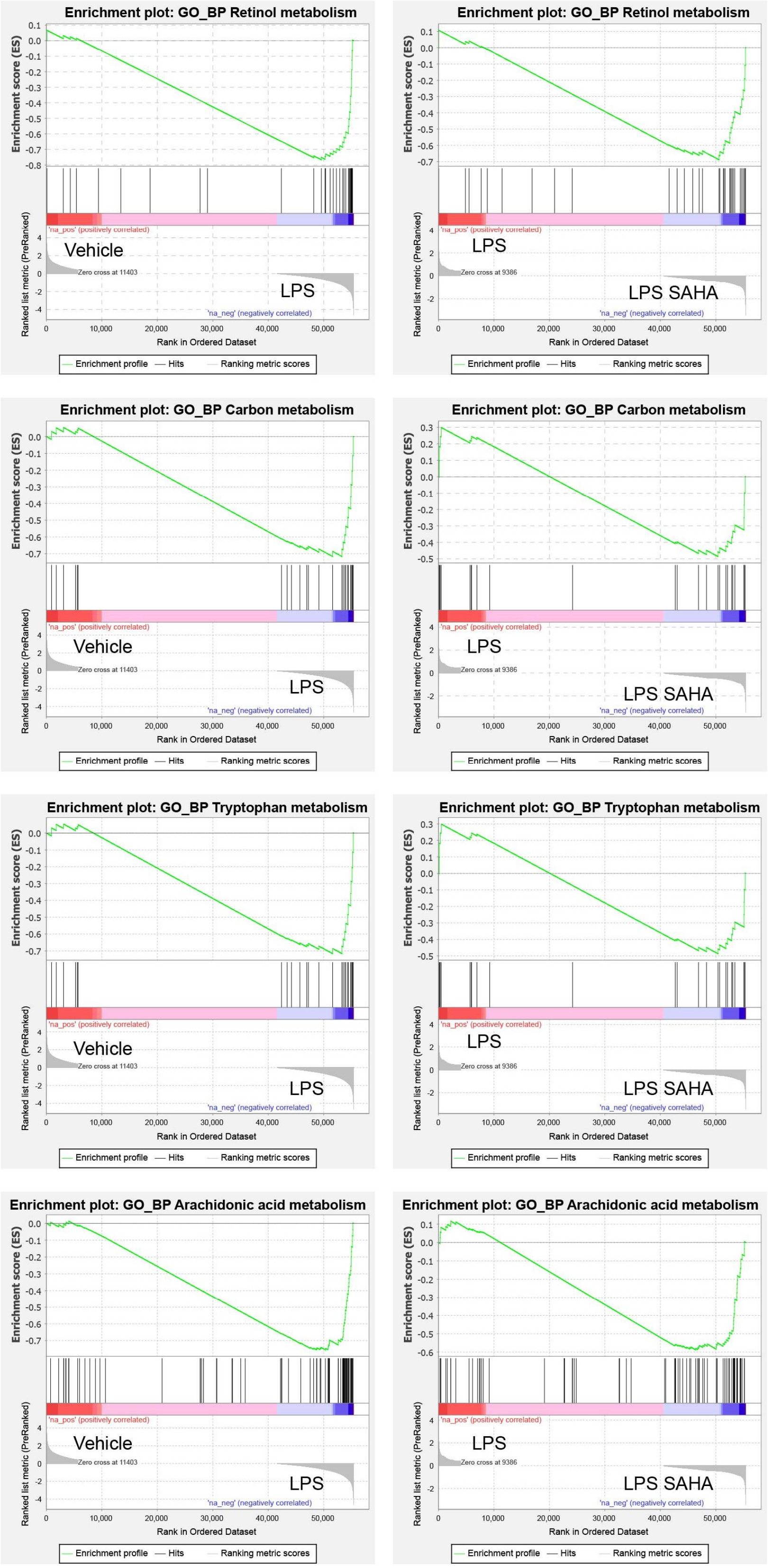
Enrichment plot: GO_BP Retinol metabolism
Vehicle
LPS
Enrichment plot: GO_BP Retinol metabolism
LPS
LPS SAHA
Enrichment plot: GO_BP Carbon metabolism
Vehicle
LPS
Enrichment plot: GO_BP Carbon metabolism
LPS
LPS SAHA
Enrichment plot: GO_BP Tryptophan metabolism
Vehicle
LPS
Enrichment plot: GO_BP Tryptophan metabolism
LPS
LPS SAHA
Enrichment plot: GO_BP Arachidonic acid metabolism
Vehicle
LPS
Enrichment plot: GO_BP Arachidonic acid metabolism
LPS
LPS SAHA
Enrichment score (ES)
Ranked list metric (PreRanked)
'na_pos' (positively correlated)
'na_neg' (negatively correlated)
Zero cross at 11403
Zero cross at 9386
Rank in Ordered Dataset
Enrichment profile
Hits
Ranking metric scores

Figure S6

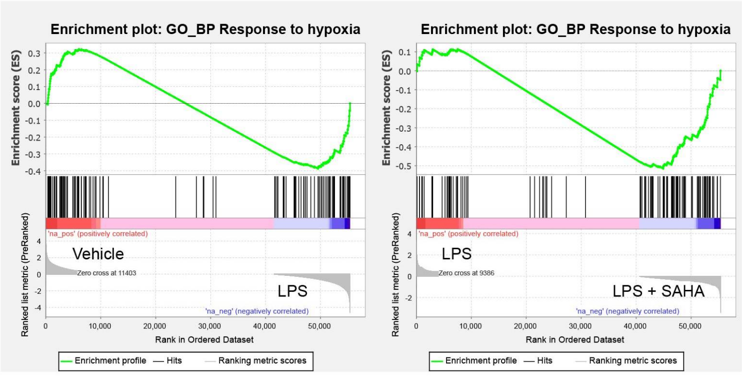

Figure S7

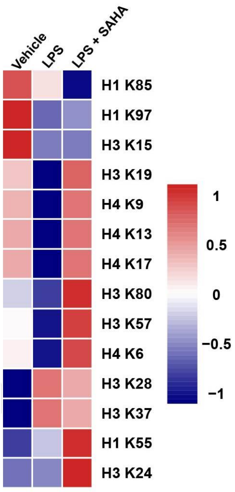

Figure S8

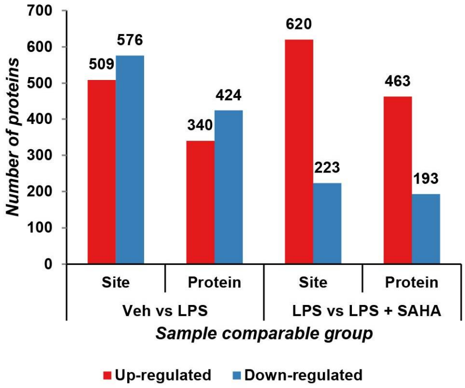

Figure S9

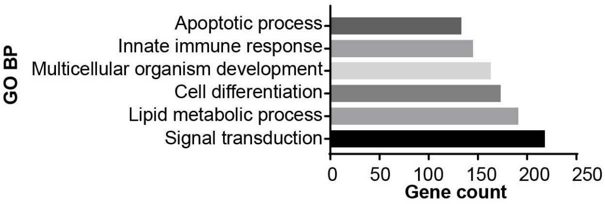

Figure S10

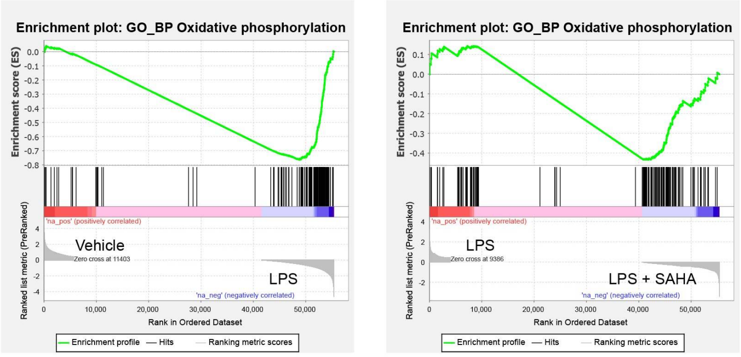

Figure S11

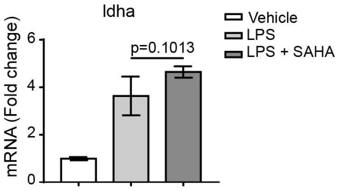

Figure S12

Sequence	Species
EEEARLKKSA	*Mus musculus*
EEEARLKKSA	*Rattus norvegicus*
EEEARLKKSA	*Homo sapiens*
EEEAHLKKSA	*Sus scrofa*
EEEARLKKSA	*Macaca fascicularis*

Figure S13

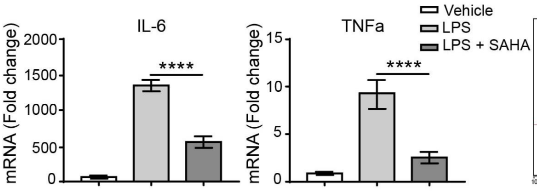

Figure S14

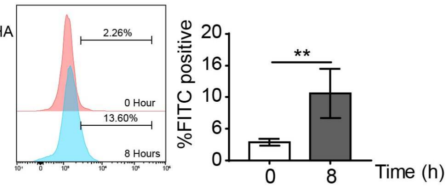

Figure S15

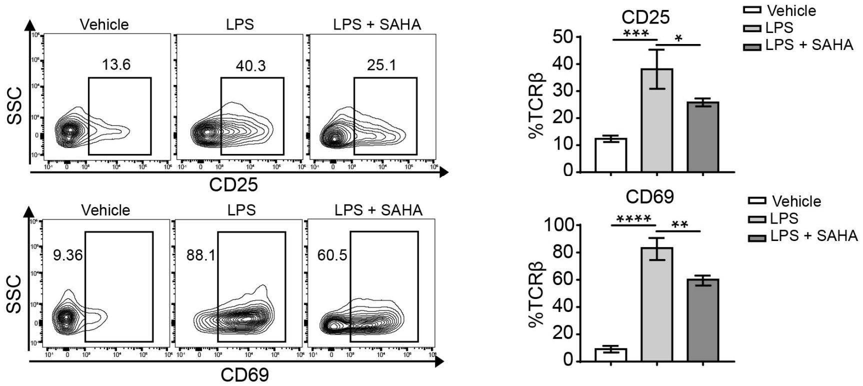

S1. Haematology analysis of the absolute count of white blood cells, lymphocytes, granulocytes and monocytes in mouse peripheral blood 8 hours after the treatment in indicated groups (n=5 animals /group) Results in S1 are shown as averages ±SD. *p < 0.05; **p < 0.01; ***p < 0.001; ****p < 0.0001, by one-way ANOVA.

S2. Multiplexed in vivo imaging determine the biodistribution of FITC-conjugated LPS (1 mg/kg i.p.) in indicated organs 8 hours post-injection (n=3 animals /group).

S3. Hematoxylin and eosin (HE) staining of liver sections in indicated groups. BS: blood sinusoids; PN: pyknotic nuclei; BN: binucleated hepatocytes; MI: monocytes infiltration; HN: hepatic cell necrosis (n=3 animals /group).

S4. The volcano plot shows differentially expressed genes (fold change>1) in LPS (30 mg/kg i.p.) versus the vehicle-treated group.

S5. GSEA analysis was applied using the hallmark genes from the MSigDB Database in indicated treatment conditions and pathways (Retinol metabolism; Carbon metabolism; Tryptophan metabolism and Arachidonic acid metabolism).
S6. GSEA analysis was applied using the hallmark genes from the MSigDB Database in the biological process of response to hypoxia in indicated treatment conditions.
S7. Heatmap showing the acetylation levels of histone acetylation sites in the indicated treatment condition.
S8. Bar chart showing the number of significantly changed protein and acetylation sites in indicated treatment conditions.
S9. Gene ontology (GO) terms enrichment analysis showed enrichment of top-ranked biological process (BP) in LPS versus vehicle-treated group.
S10. GSEA analysis was applied using the hallmark genes from the MSigDB Database on the biological process of oxidative phosphorylation pathway in indicated treatment conditions.
S11. Gene expression levels of LDHA in BMDMs were determined in indicated treatment conditions.
S12. The amino acid sequence of conserved lysine 318 site in indicated species.
S13. Analysis of IL-6, TNFa mRNA expression levels by qPCR in BMDMs with indicated treatment conditions.
S14. The proportion of FITC-positive Kupffer cells in the liver was determined with flow cytometry.
S15. T cell activation markers CD25 and CD69 staining and gated from the total T cell population (n=3 animals/group).

Table S1

**Chemicals, Reagents, Mediums**	**Source**	**Identifier**
Lipopolysaccharides from Escherichia coli O55:B5	Sigma-Aldrich	Cat# L2880
Lipopolysaccharides from Escherichia coli O111:B4	Sigma-Aldrich	Cat# F3665
Vorinostat (SAHA)	MedChemExpress	Cat# HY-10221
2-Deoxy-D-glucose	MedChemExpress	Cat# HY-13966
Percoll	Merck	Cat# P1644
Collagenase Type IV	Sigma-Aldrich	Cat# C4-BIOC
Nicotinamide (NAM)	Sigma-Aldrich	Cat# N3376
DAPI Staining Solution	Abcam	Cat# AB228549
VECTASHIELD Antifade Mounting Medium	VectorLaboratories	Cat# H-1000-10
HyClone Phosphate Buffered Saline solution (DPBS)	Cytiva	Cat# SH30256.FS
Ethylenediaminetetraacetic acid	Merck	Cat# E9884
HyClone Dulbecco's Modified Eagle Medium (DMEM) with high glucose	Cytiva	Cat# SH30022.02
Fetal Bovine Serum	Gibco	Cat#26140079
Penicillin-Streptomycin Solution	Gibco	Cat# 15140130
TRIzol™ Reagent	Invitrogen	Cat# 15596026
**Antibodies**		
Rabbit anti-Mouse IgG	Abcam	Cat# AB7000
Rabbit polyclonal anti-PYCARD Antibody	Invitrogen	Cat# PA5-50915
Rat monoclonal anti-TOMM20 Antibody	Abcam	Cat# AB309083
Mouse monoclonal anti-FLAG Antibody	Sigma-Aldrich	Cat# F3165
Rat Monoclonal anti-F4/80 Antibody	Biolegend	Cat# 123107
Rat Monoclonal anti-CD206 Antibody	Biolegend	Cat# 141707
Rat Monoclonal anti-CD25 Antibody	Biolegend	Cat# 102037
Rat Monoclonal anti-CD45 Antibody	Biolegend	Cat# 157211
Rat Monoclonal anti-CD86 Antibody	Biolegend	Cat# 159203
Rat Monoclonal anti- Ly6G Antibody	Biolegend	Cat# 127653
Rat Monoclonal anti-Ly6C Antibody	Cell Signaling	Cat# 75092
Rat Monoclonal anti-CD19 Antibody	Invitrogen	Cat# 17-0193-82

Rat Monoclonal anti-CD44 Antibody	Cell Signaling	Cat# 75122
Armenian Hamster Monoclonal anti-CD11c Antibody	Biolegend	Cat# 117321
Armenian Hamster Monoclonal anti-TCR β Antibody	Biolegend	Cat# 117321
Armenian Hamster Monoclonal anti-CD69 Antibody	Invitrogen	Cat# 25-0691-82
Rat Monoclonal anti-CD11b Antibody	Cell Signaling	Cat# 16538
Ghost Dye	Cell Signaling	Cat# 18452
Rabbit Polyclonal Anti-LDHA Antibody	Invitrogen	Cat# PA5-27406
Anti-Rabbit HRP conjugated secondary antibody	Santa Cruz	Cat# SC-2004
Anti-Mouse HRP conjugated secondary antibody	Santa Cruz	Cat# SC-2005
**Experimental Models: Cell Lines and Animals**		
Human HEK293T	ATCC	Cat# CRL-11268
Mouse L929		Cat# CCL-1
C57BL/6 mice	GD Animal Center	
**Software and Algorithms**		
SnapGene		www.snapgene.com/
AdobeIllustrator		www.adobe.com/
Gene Set Enrichment Analysis		www.gsea-msigdb.org/
FlowJo		www.flowjo.com/
R 3.6.1		www.r-project.org/
GraphPad Prism		www.graphpad.com/
ImageScope		www.leicabiosystems.com/
BioRender		www.biorender.com/
**Recombinant DNA and Oligonucleotides**		
Plasmid: pCMV-Tag 2a	Stratagene	Cat# 6084-1
Plasmid: pBluescript	Stratagene	Cat# 6084-2
Plasmid: pGFP	This study	
Plasmid: pCMV-Tag2a-LDHA	This study	
Plasmid: pCMV-Tag2a-LDHA-KQ57	This study	
Plasmid: pCMV-Tag2a-LDHA-KQ155	This study	
Plasmid: pCMV-Tag2a-LDHA-KQ232	This study	
Plasmid: pCMV-Tag2a-LDHA-KQ318	This study	
Plasmid: pCMV-Tag2a-LDHA-KR318	This study	
Mouse IL1b<br>Forward:AGTTGACGGACCCCAAAAG<br>Reverse:AGCTGGATGCTCTCATCAGG	SANGONG	
Mouse IL6<br>Forward:CCAGGTAGCTATGGTACTCCA<br>Reverse:GCTACCAAACTGGCTATAATC	SANGONG	
Mouse Tnf<br>Forward:CCCTCACACTCAGATCATCTT<br>Reverse:GCTACGACGTGGGCTACAG	SANGONG	
Mouse Ifn- β Froward:GCCTTTGCCATCCAAGAGATGC<br>Reverse:ACACTGTCTGCTGGTGGAGTTC	SANGONG	
Mouse D-loop<br>Forward:AATCTACCATCCTCCGTGAAACC<br>Reverse:TCAGTTTAGCTACCCCCAA GTTTAA	SANGONG	
Mouse 18S<br>Forward:TAGAGGGACAAGTGGCGTTC<br>Reverse:CGCTGAGCCAGTCAGTGT	SANGONG	
Mouse Tert	SANGONG	

Forward:CTAGCTCATGTGTCAAGACCCTCTT<br>Reverse:GCCAGCACGTTTCTCTCGTT	
Mouse Cox<br>Forward:GCCCCAGATATAGCATTCCC<br>Reverse:GTTCATCCTGTTCCTGCTCC	SANGONG
Mouse Non-Numt<br>Forward:CTAGAAACCCCGAAACCAAA<br>Reverse:CCAGCTATCACCAAGCTCGT	SANGONG
Mouse B2m<br>Forward:ATGGGAAGCCGAACATACTG<br>Reverse:CAGTCTCAGTGGGGGTGAAT	SANGONG
